# A texture-processing model of the ‘visual sense of number’

**DOI:** 10.1098/rspb.2014.1137

**Published:** 2014-09-07

**Authors:** M. J. Morgan, S. Raphael, M. S. Tibber, Steven C. Dakin

**Affiliations:** 1 Max Planck Institute for Neurological Research, PO Box 41 06 29, Cologne 50866, Germany; 2 Institute of Ophthalmology, University College London, Bath St., London EC1V 9EL, UK

**Keywords:** psychophysics, numerosity, texture perception

## Abstract

It has been suggested that numerosity is an elementary quality of perception, similar to colour. If so (and despite considerable investigation), its mechanism remains unknown. Here, we show that observers require on average a massive difference of approximately 40% to detect a change in the number of objects that vary irrelevantly in blur, contrast and spatial separation, and that some naive observers require even more than this. We suggest that relative numerosity is a type of texture discrimination and that a simple model computing the contrast energy at fine spatial scales in the image can perform at least as well as human observers. Like some human observers, this mechanism finds it harder to discriminate relative numerosity in two patterns with different degrees of blur, but it still outpaces the human. We propose energy discrimination as a benchmark model against which more complex models and new data can be tested.

## 1. Introduction

**Figure 1.**
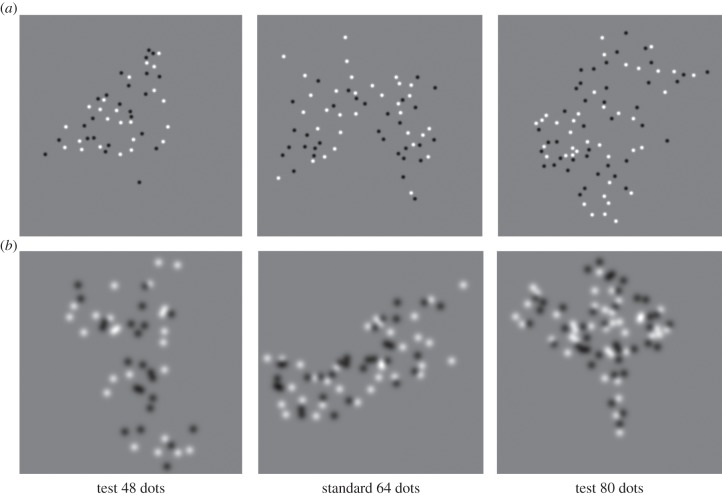
Examples of stimuli used in the experiments to measure the accuracy of relative numerosity discrimination. (*a*) Dots blurred with *s* = 2 pixels. (*b*) The case of *σ* = 6 pixels. In the equal-blur condition, both the test and standard had *s* = 2 pixels. In the unequal-blur condition, the blur for the test was chosen randomly on each trial in the range 2–6 pixels, as was that of the standard.

If the dots in figure 1 were fruits on a tree, there would be obvious advantages to a foraging animal in perceiving at a glance which tree had the most fruits. Not surprisingly, then, there are many demonstrations of relative numerosity discrimination in animals and humans. Relative numerosity discrimination has been studied experimentally in adults [1–4], infants [5,6] and non-human species [7–9], using psychophysics, fMRI [10,11] and single unit physiology [12]. The mechanism for relative numerosity discrimination has proved elusive [13], in part because of the inevitable correlations between number and ‘irrelevant’ stimulus parameters such as overall pattern size, density and size of the elements. An ideal numerosity mechanism would not care about the shape and spatial distribution of objects in the scene. However, it is known that perceived numerosity can be influenced by many properties of the objects, such as their size, density and spatial arrangement [13–15]. The problem we face at present is that there is no simple standard model of numerosity computation against which to test these empirical findings. We suggest that debates and *Gedankenexperimente* on this issue are pointless in the absence of a computable model of relative numerosity discrimination against which data can be tested. Even an incomplete model would be better than none at all. Here, we describe such a model, based on contrast energy [16], and compare its performance with that of the human observer.

The intuition behind the model is easy to grasp. As we add more objects to an image we add more contour. The amount of contour can be estimated from the combined output of ‘edge detectors’ that respond to local changes in luminance. To make these detectors sensitive to the difference between one object and two occupying the same area, and to be insensitive to their spacing, we want the detectors to be as small as possible. In physiological terms, this means using small ‘receptive fields’; in Fourier-optical terms, it means measuring the energy at high spatial frequencies. We therefore measure the energy in our images at high spatial frequencies and use this as a proxy for numerosity. We expect this model to make mistakes if we vary object attributes such as their size, density and spatial-frequency content. For example, randomly blurring the objects will decrease their high-spatial-frequency content without necessarily affecting their number. However, rather than dismissing the model *a priori* on these grounds we ask: ‘How much does blur degrade the performance on the model, and how does this compare with the performance of a real human observer?’ Only if we find that the human observer is better than the model do we consider adding further complexity to the model such as multiple frequency channels [13].

We measured observers' ability to distinguish patterns differing in numerosity (figure 1) using a temporal two-alternative forced choice (2AFC) design in which a standard stimulus containing 64 dots occupying a constant area but with irregular shape was presented on each trial along with a test stimulus containing either fewer or more dots. Each of the dots was blurred with a two-dimensional Gaussian filter (see Material and methods).

While the number of dots was always different in the test and standard stimuli, on half the trials the test stimulus differed in dot density with area held constant, whereas on the remaining trials area varied while density remained constant [1]. Because numerosity just-noticeable differences (JNDs) tend to follow Weber's law of proportionality, we expressed discrimination ability as the Weber Fraction (JND × 100/64).

## 2. Results

### (a) Experiment 1

In the equal-blur condition illustrated in the top row of figure 1, all the dots had the same blur (*σ* = 2 pixels). In the unequal-blur condition, the dots in the standard and test stimuli were independently blurred with *σ* in the range 2–6 pixels. The bottom row of figure 1 shows stimuli blurred with the maximum blur of *σ* = 6 pixels. The equal- and unequal-blur conditions were run in separate blocks of 128 trials to find the JND in numerosity between test and standard.

**Figure 2.**
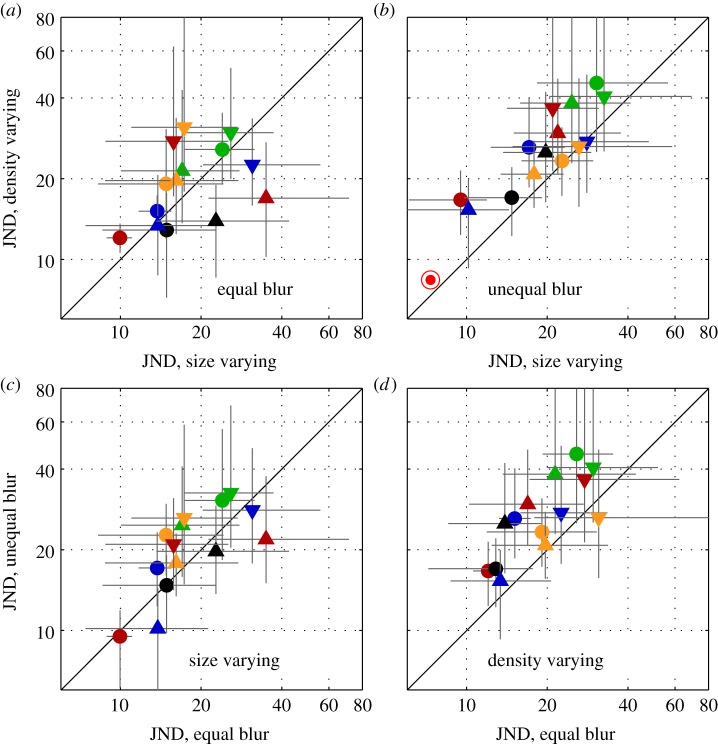
Each panel shows the JNDs in numerosity for 84% correct discrimination, with different combinations of symbol shape and colour for each subject. The red bullseye symbol in panel (*b*) shows the performance in the unequal-blur condition of the model observer described in the text. The error bars represent 95% CIs.

Our data showed large individual differences in subjects' ability to discriminate differences in numerosity (figure 2). The best subjects in the best condition had Weber fractions less than 10% and the worst in the same condition as high as 35%. Pairwise correlations between conditions (table 1) showed that subjects who were good in one condition tended to be good in all conditions. Performance was also worse in some conditions than others. The worst performance was in the density-varying, unequal-blur condition, where the mean Weber fraction was 27.8%. Pairwise *t*-tests revealed significant differences in all three cases involving the density-varying, unequal-blur condition (size-varying, equal-blur versus density-varying, unequal-blur, *p* = 0.0038; density-varying, equal-blur versus density-varying, unequal-blur, 0.0076, size-varying, unequal-blur versus density-varying, unequal-blur, *p* = 0.0005), All these differences are significant at the Bonferroni-corrected significance level of 0.0083. No other pairwise differences were significant. The poorer performance in the unequal-blur case could be due either (i) to performance being poorer at large blurs, (ii) to unequally blurred stimuli being difficult to compare for numerosity or (iii) to the general decrement in acuity when different conditions are randomly interleaved [17]. To distinguish these possibilities, we reanalysed the unequal-blur condition separating out those trials when the test and standard had the same blur from trials when the blur was the same. There was no significant difference between these sub-sets. Nor were there any systematic or significance differences due to level of blur when the test and standard had the same blur. The most probable reason for the effect of unequal blur is thus a general psychophysical decrement due to the interleaving of different conditions.

**Table 1.** Pairwise correlation coefficients (Pearson's) between the performances of subjects in the four number discrimination tasks, with size or density (dens) varying and blur equal (eq) or unequal (uneq).

	size-eq	dens-eq	size-uneq	dens-uneq
size-eq		0.29	0.63	0.45
dens-eq			0.81	0.71
size-uneq				0.82

To model the data, we consider relative numerosity as a form of texture processing, and use what Chubb & Landy [18] call a ‘back pocket’ model of texture discrimination. Images of the stimuli seen by the human observers were clipped to the stimulus size and filtered, and the energy difference between standard and test on each trial was used to generate a decision (see Material and methods). We stress that the model decisions were made on a trial-by-trial basis, not on averages. Thus, the model observer had no more and no less information than the real human observer.

We follow Dakin *et al*. [13] in measuring the energy of the patterns in two spatial-frequency passbands, derived from Laplacian-of-Gaussian filters tuned to high (*σ* = 2 pixels) or lower (*σ* = 8 pixels) spatial frequencies. The intuition here is that numerosity is encoded by the amount of ‘detail’ in the image, which is well captured by its high-spatial-frequency content. Indeed, the energy captured by the high-spatial-frequency filter in the case where the test and standard have equal blur discriminates relative numerosity virtually perfectly (JND < 1%), whereas a low-spatial-frequency filter does so about as well as the average human observer (JND 15% for size-varying and 20.5% for density-varying conditions, respectively). The reason why the low-spatial-frequency filter is less reliable is because the random outline shape of the pattern perturbs it, as was our specific intention in designing the stimuli.

**Figure 3.**
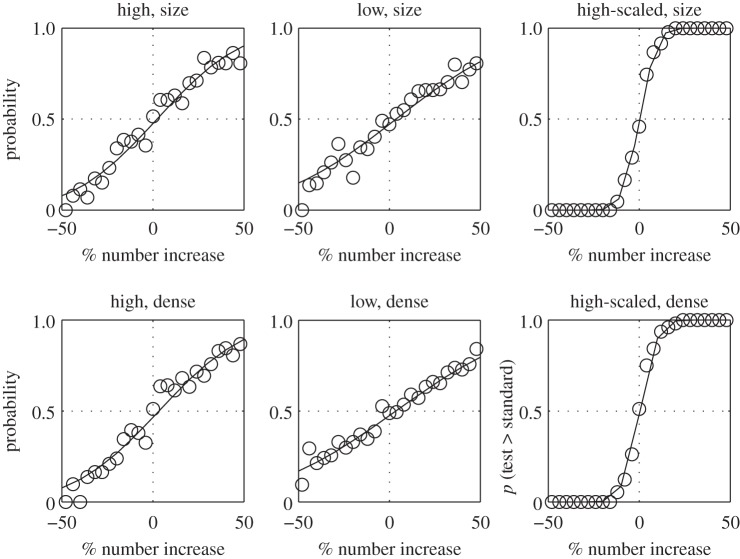
Each panel shows a psychometric function based on the trial-by-trial decisions of the model observer given the actual stimulus pairs of unequally blurred stimuli presented to the human observers. The key above each panel indicates whether the model was based on the high, low or high-scaled passbands and whether the stimuli differed in area (size) or density (dense). The high-scaled condition scaled the high-frequency energy by the amount of blur in the stimulus, independently calculated by the MIRAGE algorithm. For further details see the text.

However, as we had also anticipated, the high-spatial-frequency filter copes relatively poorly with unequal blur between the stimuli. The psychometric functions produced from the model observers are shown in figure 3.

JNDs were 37.98% and 37.62% for size and density conditions, respectively. This is worse than the best human observers, though better than some. The low-spatial-frequency filter is even worse (51.5 and 56%).

Poor performance of the high-spatial-frequency filter with blur mismatch is understandable. Different levels of blur alter the spatial-frequency content of a stimulus—and the response of a filter by different amounts—rendering a comparison of two filter responses unreliable. To enable the high-spatial-frequency filter to do better, we scaled its output by the amount of blur in the stimulus. To determine image blur, the model observer isolated single dots and measured the blur with the MIRAGE algorithm [19], which encodes blur as the distance between the zero-bounded regions in the second spatial derivative. Using MIRAGE and a second-order polynomial fit, we determined the empirical relationship between blur (*s* in arcmin) and contrast energy in the highest-spatial-frequency channel to be as follows: log(*E*) = 0.0021*s*^2^ − 0.057*s* + 13.06.

This relationship was used to normalize the contrast energy so that it was independent of blur. Figure 3 shows that normalization allowed a more accurate prediction of numerosity, producing JNDs of 7.34% and 7.87%, respectively—better than any of the human subjects.

### (b) Experiment 2

**Figure 4.**
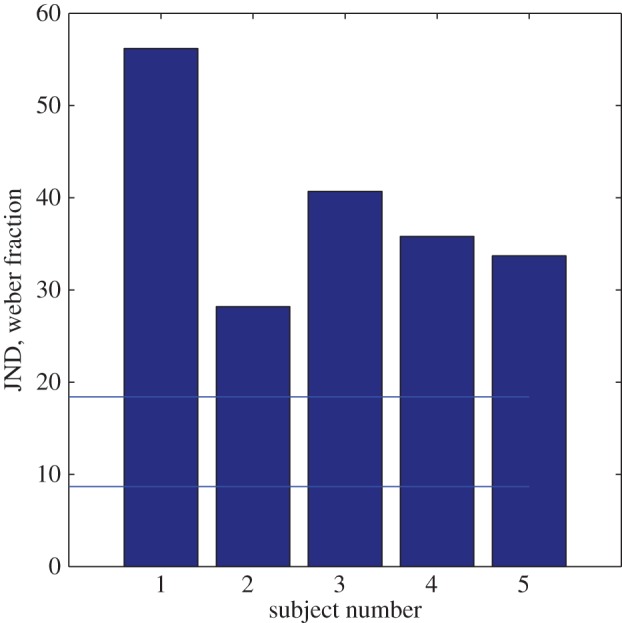
Five observers' numerosity discrimination performance in the ‘kitchen sink’ experiment where stimuli contained irrelevant perturbations of element contrast, size and blur. Each bar shows the Weber fraction for a single subject. The lower of the two horizontal lines shows performance of a model observer with the same stimuli, when contrast variation was not included. The higher line shows performance with contrast variation and logarithmic contrast compression.

It is known that approximate number discrimination, measured by the Weber fraction, can be affected by image properties other than number (e.g. [20]) but it is not known how high the Weber fraction can be if different sources of image variation are combined. To determine this, we combined different sources of variability each of which would be expected to affect the spatial-frequency content of the stimuli. In a ‘kitchen sink’ experiment, we varied (i) the blur of each of the elements independently within each display (rather than keeping it constant, as in the previous experiment); (ii) the size of the test and standard, independently in the range 1 : 2*S*, where *S* was the area of the standard in the previous experiment; and (iii) the contrast of all the elements in the display, independently for test and standard over a range from 0.13 to unity (see Material and methods). All the elements remained visible. We also looked at the case where there was no contrast variation. The test always contained more dots than the standard and the method was still 2AFC. The mean Weber fraction over five subjects (figure 4) was 38.92%. There was no significant difference between contrast-varying and contrast-constant thresholds. The same set of images was shown to the model observer. Without contrast variation, Weber fractions for the high-frequency channel were less than 10%, considerably better than the human observers. However, as we had anticipated, contrast variation made the task impossible for the model, whereas it had little effect on the human observer [15]. To rescue the model, we took account of compression of the transduced signal by contrast gain control [21]. Specifically, we reduced the range of contrasts in the range of the experiment logarithmically. This reduced the Weber fraction for the model observer to 17%, better than that of the human observers (figure 4).

### (c) Experiment 3

**Figure 5.**
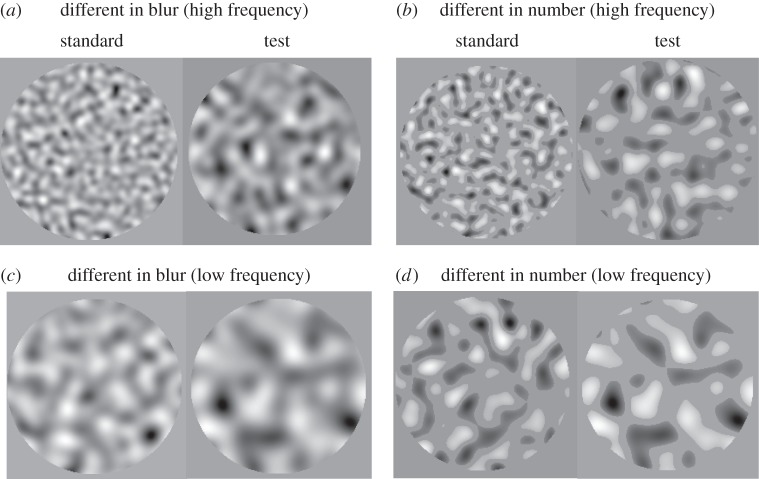
Stimulus pairs used to measure subject's ability to discriminate differences in either (*a*,*c*) blur or (*b*,*d*) discrete blob number. The frequency content of the standard stimulus was either (*a*,*b*) high or (*c*,*d*) low. The members of each pair were presented sequentially with the test and standard in random order.

**Figure 6.**
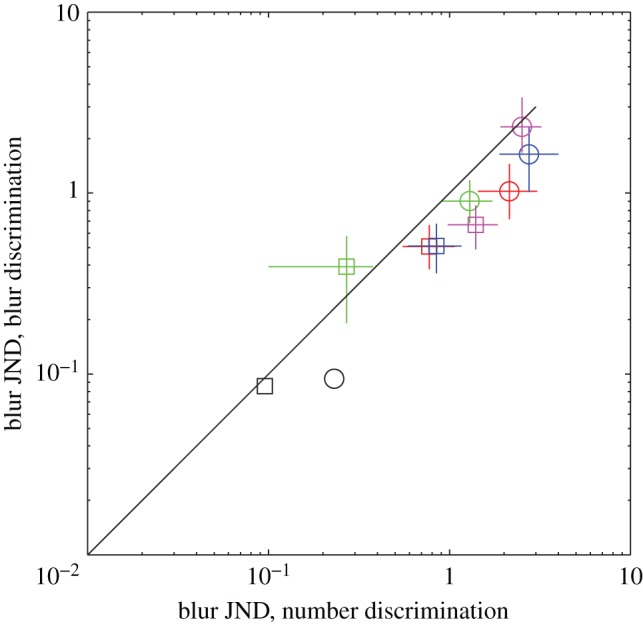
Just-noticeable differences (JNDs) for blur discrimination (vertical axis) plotted against those for blob number discrimination (horizontal axis), for four different observers (differently coloured symbols). Circles and squares show results for relatively low-frequency standard pedestals and relatively high-frequency stimuli, respectively. The two black symbols show performance of the Watson–Ahumada energy model with the same stimuli as those seen by the human observers.

When an image containing many closely spaced objects is blurred, the objects coalesce and their number is reduced. Thus, a change in blur could be alternatively described as a change in numerosity. It would be interesting to measure whether thresholds for blur discrimination, measured in units of a blurring function, are similar to those for number discrimination when described as a Weber fraction for number. If this proves to be so, it would strengthen the connection between discrimination of number and of other visual properties of the image. To test this idea, we carried out a further experiment in which subjects attempted to discriminate between pairs of stimuli illustrated in figure 5. The stimuli were derived by blurring white pixel noise with a difference-of-Gaussian filter. Observers carried out two different tasks in different blocks of trials. In the blur discrimination case, they decided which of the two stimuli (standard and test, in random order) was more blurred. In the number discrimination case, the same stimuli were thresholded (i.e. grey levels less than 1 s.d. from the mean were set to the mean grey level) to split them up into discrete blobs (figure 5*b*,*d*) and observers decided which stimulus contained the more blobs. In both cases, we determined the JNDs in the space constant of the blurring filter by the psychophysical method described earlier. The data show that contrast energy thresholds for the two tasks were similar, with a general trend for thresholds to be higher in the number case. Note that this last difference does not imply different mechanisms for number and blur, because information has been reduced from the number stimuli by thresholding. To model the results, we used the Watson–Ahumada energy model of blur discrimination [16], which computes the energy in the stimulus after passing the stimulus through a filter representing the contrast sensitivity function of human vision (figure 6). Although much better than the human observer at the task given exactly the same stimuli, the model captures the similar contrast energy thresholds for blur and numerosity discriminations, and the slightly lower threshold for blur than number. Moreover, when JNDs in the number discrimination case were recalculated as differences in blob number rather than blur, the mean Weber fraction of 23% fell right in the middle of the range for traditional numerosity.

### (d) Experiment 4

**Figure 7.**
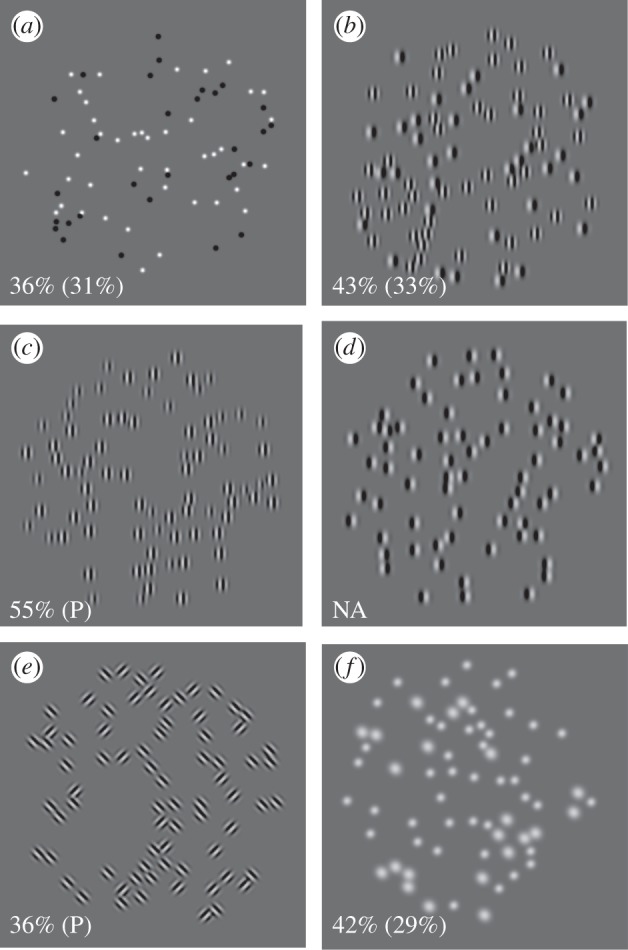
Various configurations for discriminating relative numerosity of two classes of element in the same texture. Each panel is labelled with the Weber fraction of one observer (M.J.M.) along with the (bracketed) prediction of a filter energy ratio model. The cases are (*a*) contrast polarity, (*b*) spatial frequency (×2), (*c*) contrast (×2), (*d*) phase (90°), (*e*) orientation (90°) and (*f*) size (×2). In the case of phase, the psychometric function was flat. The numbers in the bottom left of each panel indicate the observer's Weber fraction followed by that of the model. The key ‘P’ in panel (*e*) indicates that model performance was perfect.

Next, we consider the case of relative numerosity in single textures. It is known that pigeons [22] and human subjects [23,24] can decide which of two kinds of element in a mixed texture is the more numerous, albeit sometimes with strong biases towards one of the element classes [23,24]. An example is the ratio of black to white dots (figure 7*a*). This ability would demand a multi-channel model rather than the single-channel model we have used previously. To determine which channels might be available, we tested a single observer (M.J.M.) with the six kinds of mixed texture in figure 7. To prevent a single channel being used, the total number of dots was varied randomly over trials ((64 + *x*), where *x* was from the uniform distribution 0–21 dots). Weber fractions varied from 36% for mixed polarity or orientation to 70% for mixed size. The case of mixed phase was impossible (as the reader can see in the figure), suggesting a link with the literature on ‘pop-out’, where phase is not a salient feature [25].

The values in brackets after the observer performance are the Weber fractions for a model observer classifying the same stimuli, using the ratio of energy in two channels on each trial and comparing to the mean ratio in the set of stimuli seen before that trial. In the case of dot polarity (figure 7*a*), the channels were half-wave rectified [26–28], high-spatial frequency. In figure 7*b*, the channels were two isotropic spatial frequency tuned channels two octaves apart (2 and 8 pixels space constant). In figure 7*c*, a single channel was used but thresholded at two different levels to isolate the two kinds of dot. Modelling of figure 7*d* was not attempted because the observer finds the task impossible. Figure 7*e* was analysed with two orientation-tuned channels 90° apart and figure 7*f* was analysed with the same two channels as in figure 7*b*.

### (e) Experiment 5

**Figure 8.**
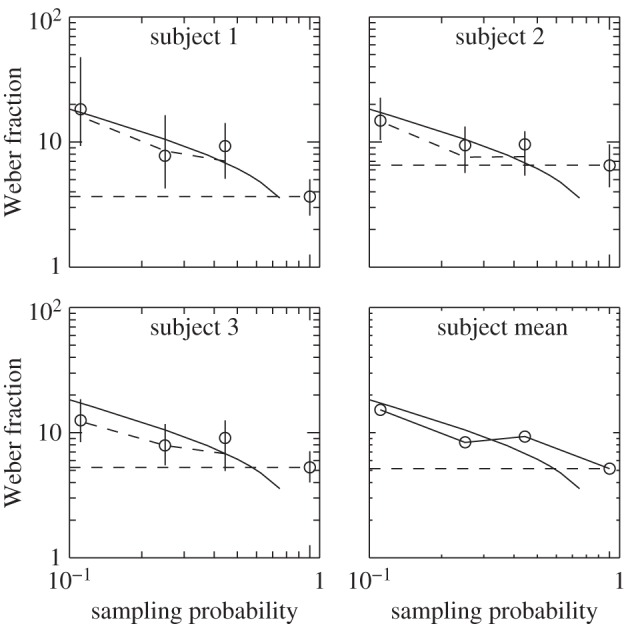
Results of Experiment 5, in which observers (subjects 1–3) decided whether there are more black than white dots in a display such as figure 7, with a circular mask in front of the display so that only a central circular area was actually visible. The size of the aperture was systematically varied and the observer's accuracy measured as in previous experiments. Weber fractions (vertical axis) for the real observers are shown by circles with 95% confidence intervals (vertical error bars). The continuous curve shows how we would expect the performance of the ideal observer to improve (from left to right in the figure) as we increase the proportion of the 64 dots in the whole pattern actually presented to the observer (horizontal axis). The broken curve shows the performance of a simulated observer presented with the same images as the real observer. By drawing the horizontal line shown in the figure, we can determine that the real observer presented with 64 dots does as well as an ideal observer shown approximately half that number. (Online version in colour.)

It is well established that normal subjects can make errorless estimates of number in the ‘subitizing’ region of one to six dots [29], so a possible mechanism for relative numerosity is to take an equivalent area of the two patterns sufficiently small to include a number in the subitizing region and count the dots therein. To examine this possibility, we used the task illustrated in figure 7*a* of deciding whether there are more black than white dots, and placed a circular mask in front of the display so that only a central circular area was actually visible. In order not to disadvantage the real observer relative to the ideal and to simplify calculation of ideal performance, dot overlap was prevented by placing an exclusion zone around each dot, and the total number of dots was kept constant at 64. The size of the aperture was systematically varied and the observers' accuracy measured as in previous experiments. Three observers were used. The observers' performance was compared with that of an ideal observer that could count the number of dots within the viewing aperture without error. Of course, this observer necessarily makes an increasing number of errors as the aperture size is reduced, because the actual number of black and white dots has random (binomial) sampling error. The red curve in figure 8 shows how we would expect the performance of the ideal observer to improve (from left to right in the figure) as we increase the proportion of the 64 dots in the whole pattern actually presented to the observer (horizontal axis). The real observer (circles) also benefits from increasing sample size, but never gets be as good as the ideal. By drawing the horizontal line shown in the figure, we can determine that the real observer presented with 64 dots does as well as an ideal observer shown about half that number. Therefore, we can conclude that whatever the mechanism used by the real observer for relative numerosity, it is no worse or better than if it randomly selected 50% of the dots and counted them accurately. As this number in the present case is 32, we can decisively rule out the ‘subitizing’ explanation of relative numerosity accuracy.

## 4. Discussion

These experiments were not designed to rule out the existence of a mechanism for discrete numerosity discrimination, nor indeed could any finite set of experiments prove a negative. On the contrary, our experiments demonstrate that human observers are able to make estimates of numerosity despite large changes in image properties such as blur and contrast. On the positive side, we have shown that human performance can be matched, or exceeded, by a mechanism for contrast energy discrimination that incorporates scaling for changes due to contrast and blur, and which can flexibly take into account energy in different passbands of orientation and frequency. Whether we call this a ‘special’ mechanism for numerosity or another example of flexible pattern recognition is not addressed by our findings. We suggest that further computational investigations are more important than semantic issues.

It is sometimes said that human subjects have ‘no difficulty’ with relative numerosity tasks [30], but this statement has little meaning unless a metric for comparison is defined. One such metric is the Weber fraction, which is the proportional change in stimulus magnitude that can be detected at a criterion level such as 80% correct. For luminance, and for vernier acuity based on the light distribution, the Weber fraction is approximately 2% [31]. For size, distance and area of regular shape, it is 5–10% [32,33]. Against these standards numerosity is rather poor. Fractions as low as 10% are found only when other cues such as area are available [14]. We have shown here that values of 30% are more typical when the use of alternative cues is prevented and that some observers can have values as high as 50%. Another way of measuring the accuracy of relative numerosity discrimination is to quantify its statistical efficiency, and we have shown (experiment 5) that this is no higher than if the observer sampled only 50% of the dots in a 64-dot display. As it is unlikely that the observer literally sub-samples before counting, we should consider other mechanisms from counting to explain performance. There are abundant demonstrations that numerosity estimation is affected by low-level image properties [2,3,13,15,20,34–36]. In these circumstances, it seems to make sense to look for a variety of heuristics that the observer can use, rather than some specialized ‘number sense’. ‘Back pocket’ models of texture discrimination [18] are the obvious resource.

We do not claim that contrast energy is the only mechanism available to human observers for numerosity computation [13–15]. It seems likely that there are many strategies available to the human observer for such a complex task as visual numerosity. However, our proposed model can usefully serve as a benchmark when a particular manipulation affects numerosity discrimination and we want to know whether the effect can be accounted for by changes in contrast energy. For example, it has been shown that decisions about number are disrupted when the area occupied by the dots is also varied, a result that Nys & Content describe in terms of a cognitive interference between two different quantities [37]. They did not consider the possibility that the two quantities interfered at a basic sensory level (for example, in effects on contrast energy). A simple benchmark model would be useful in such cases for determining whether a resort to higher cognitive mechanisms is necessary. It may be objected that our model requires scaling of energy by blur, and thus a degree of *a priori* knowledge by the observer. However, numerosity in this respect may be no different from many (perhaps all) other perceptual computations, such as size, where retinal size is scaled by distance [38]. It would be unusual if the computation of number did not depend on multiple sources of information [13–15].

## 5. Material and methods

### (a) Stimuli and apparatus

Except for those in figure 5, stimuli were presented on the LCD display of a MacBookPro laptop computer with screen dimensions 33 × 20.7 cm (1440 × 900 pixels) viewed at 0.57 m so that 1 pixel subtended 1.25 arcmin visual angle. The background screen luminance was 50 cd m^−2^. Stimulus presentation was controlled by Matlab and the PTB3 version of the Psychtoolbox [39,40]. Stimuli were viewed binocularly through natural pupils with appropriate corrective lenses for each subject (if normally worn for reading). The stimuli in figure 4 were monocularly viewed through a 1 mm artificial pupil and presented at 150 cm viewing distance on a Viewsonic PF817 CRT display, with pixel resolution 1024 × 768, refresh rate 140 Hz and mean luminance 33.5 cd m^−2^, controlled by a Cambridge Research Systems Visage box and software.

### (b) Subjects

The 14 subjects in the main experiment (six female) all had science degrees and varied in age from 18 to 70. Five subjects, including the four authors, had previous experience in number psychophysics; the others were naive, although they all had previous experience in other psychophysical experiments. The subjects in experiment 2 (variable blur, shape and contrast) were four from experiment 1 and one additional naive observer (O).

### (c) Stimuli and tasks

Examples of stimuli are shown in figure 1. The dots were black (0.4 cd m^−2^) or white (300 cd m^−2^) with equal probability. These dots were randomly scattered within a notional polygon generated by an algorithm that randomly varied the position and number of vertices in the polygon in each trial, and which minimized overlap between the dots. The standard stimulus contained 64 dots in area 50 000 pixels. The test stimulus contained 64 ± 64 *W* dots, where *W* is a fraction between 0 and 100% in steps of 5%. *W* was determined by an adaptive procedure (see below). The stimuli were presented for 0.5 s each in random order. The area of the test was either the same as the standard (density-varies condition) or was adjusted so that test and standard had the same density (area-varies condition).

To blur the stimulus, the dots were convolved, using the Matlab Image Processing Toolbox, with a two-dimensional Gaussian blurring function
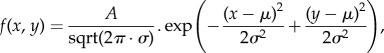
where *A* was the amplitude, set to give a contrast of 0.4 when *s* = 2; *x* and *y* were the positions relative to the centre *m*, and *s* was the standard deviation of the blurring function. As the formula shows, the contrast energy of the dots was independent of blur, but peak amplitude scaled downwards with blur. This meant that in the experiment where contrast varied randomly, the available range was 0.4–1.0 for the least blurred dots and 0.13–0.66 for the most blurred.

There was a 0.75 s blank period before each stimulus, during which only a fixation point was presented at the centre of the screen. The test and reference positions were separately offset from the fixation point to avoid interference by afterimages and to prevent the observer from using landmarks on the screen for size judgements. The offset was randomly selected in both *x* and *y* direction from a uniform distribution with a width equal to ±0.75 of the circle radius.

For five subjects, thresholds and mean values of the psychometric function for discrimination were determined by an adaptive procedure [41] designed to obtain the two parameters *μ* and *σ* (which are the 50% point and standard deviation, respectively) of the psychometric function efficiently by concentrating values of the test at ±*σ* of the psychometric function based on the data collected in previous trials. For the remaining subjects, the sequence of stimuli was identical to that generated by one of the five subjects, and their responses had no influence on the stimulus sequence. The same stimulus sequence was used to test the model.

Confidence limits (95%) for the individual points on the psychometric functions were calculated from the binomial distribution. Those for the fitted parameters of the psychometric functions were obtained by a bootstrapping procedure. The maximum-likelihood values were used to generate 160 new psychometric functions by simulation of the exact experimental procedure, and the central 95% of the fitted values were taken to define the confidence limits.

## Notes

All Psychophysical experiments with human observers were approved by the local ethics committee of the School of Health Sciences at City University London and were in conformity with the ethical principles of the Declaration of Helsinki.
